# Phylogenetic analyses to uncover the evolutionary relationship of a newly sequenced mitochondrial genome from an Eastern spinebill (*Acanthorhynchus tenuirostris*)

**DOI:** 10.1080/23802359.2020.1810149

**Published:** 2020-08-26

**Authors:** Subir Sarker, Ajani Athukorala, Saranika Talukder, David N. Phalen

**Affiliations:** a Department of Physiology, Anatomy and Microbiology, School of Life Sciences, La Trobe University, Melbourne, Australia; b School of Agriculture and Food, Faculty of Veterinary and Agricultural Sciences, The University of Melbourne, Melbourne, Australia; c Sydney School of Veterinary Science, Faculty of Science, University of Sydney, Sydney, Australia

**Keywords:** Phylogeny, avian mtDNA, order Passeriformes, family Meliphagidae, *Acanthorhynchus tenuirostris*

## Abstract

The Eastern spinebill (*Acanthorhynchus tenuirostris*), a passerine bird in the family *Meliphagidae* (honeyeaters), a dominant group of birds in Australia and New Guinea. The aim of this study was to sequence the complete mitochondrial genome of the Eastern spinebill and use its sequence to better define the phylogeny of this species. The complete mitogenome sequence of *A. tenuirostris* was circular and 16,614 bp in length, and its architecture was conserved in comparison to other mitogenome sequences under the family *Meliphagidae*. The Eastern spinebill mitogenome had the highest sequence identity with mitogenome sequences of two other honeyeaters, the white eared honeyeater, *Nesoptilotis leucotis,* (84.9%) and the white-plumed honeyeater, *Ptilotula penicillata* (85.5%). The maximum-likelihood topology distinctly discriminated the Eastern spinebill sequence against all other species of the *Meliphagidae* with significant bootstrap supports. We suggest the widespread sampling and complete mitogenome sequencing would be valuable in establishing the most accurate phylogenetic taxonomy of the family *Meliphagidae*.

## 1. Introduction

The family *Meliphagidae* (honeyeaters) is species-rich (38 genera and 180 species), morphologically diverse, and widely distributed group across Australia, New Guinea, and oceanic islands across Wallacea and the Pacific (Driskell and Christidis 2004; Andersen et al. 2019) In certain habitats, more than 12 species of honeyeater can co-occur seasonally (Keast 1985). They can be either nectarivorous, insectivorous, frugivorous, or more commonly, a combination of nectar- and insect-eating (Lea and Gray 1936).

Despite ecological and evolutionarily significance of the *Meliphagidae* the phylogenetic relationships for some species within this large family remain unclear (Andersen et al. 2019). The Eastern spinebill (*Acanthorhynchus tenuirostris*) is a honeyeater found in the woodlands of Eastern, Southeast and Tasmania Australia. Using a nuclear gene and selected mitochondrial genes, the Eastern spinebill and the Western spinebill (*Acanthorhynchus superciliosus*) have been found to form a sister clade to the remainder of the *Meliphagidae* (Driskell and Christidis 2004; Gardner et al. 2010; Andersen et al. 2014). A recent study on genus-level phylogeny of the honeyeaters using 4397 ultraconserved elements from 57 species demonstrated that *Acanthorhynchus* spinebill was sister to all remaining clades (A–H), but a completely resolved phylogeny of honeyeaters based on genome-wide data remains elusive (Andersen et al. 2019). Therefore, we believe that the complete mitogenome could play a significant role to provide more clear evolutionary relationships, the divergence time of speciation and influencing management decisions of species.

## 2. Materials and methods

### 2.1. Source of sampling and extraction of DNA

Droppings were collected from a holding bag of an Eastern spinebill caught in a mist net as part of a fauna survey in the Windsor Downs Reserve Nature (33°39′0.42′S, 150°48′31.97″E), New South Wales (Australian Bird and Bat Banding Scheme (ABBBS) Banding Authority Number 1893, ABBBS project approval—cooperative project 8529, New South Wales National Parks and Wildlife Service—Scientific License No. SL101929). The total genomic DNA was extracted using a commercial kit (Purelink™ Genomic DNA Mini Kit, Invitrogen, California, CA, USA) according to the manufacturer’s instructions.

### 2.2. Library construction and sequencing

The library preparation and sequencing were performed as previously described (Sarker et al. 2017; Sarker et al. 2018; Sarker et al. 2020). Briefly, the paired-end library with an insert size of 150 bp was prepared using the QIAseq FX DNA Library Kit (Qiagen) starting with ten ng of total genomic DNA (gDNA). The amplified library was cleaned to remove PCR-generated adaptor-dimers using JetSeq™ Clean beads (Bioline) according to the protocol described in QIAseq FX DNA Library Kits. The quality and quantity of the prepared library was assessed using an Agilent Tape Station (Agilent Technologies) by the Genomic Platform, La Trobe University. The prepared library was normalized and pooled in equimolar quantities. Cluster generation and sequencing of the pooled DNA-library were sequenced on Illumina^®^ NextSeq500 platform according to the manufacturer’s instructions (Australian Genome Research Facility, Melbourne).

### 2.3. Assembly protocol

Sequencing data used in this study were analyzed according to a previously established pipeline (Sarker et al. 2017; Sarker et al. 2019a; Sarker et al. 2019b) using Geneious (version 10.2.2, Biomatters, New Zealand) and CLC Genomics Workbench (version 9.5.4). Briefly, a total of 11.7 million reads with a read length of 150 bp was used to obtain the complete mitochondrial genome of Eastern spinebill. Preliminary quality evaluation for all raw reads was generated, pre-processed to remove ambiguous base calls and poor-quality reads, and trimmed to remove the Qiagen Universal adapter sequences. The trimmed reads that passed the quality control based on PHRED score were used as input data for *de novo* assembly using SPAdes assembler (version 3.10.0) (Bankevich et al. 2012) in Geneious. Annotation was performed using default parameter under the genetic code of vertebrate mitochondrial (transl_table 2) in Geneious (version 10.2.2). 

### 2.4. Phylogenetic analysis

Phylogenetic analyses were performed using the mitogenome sequence of Eastern spinebill determined in this study with other selected mitogenome sequences belong to the order *Passeriformes* in the GenBank database. Mitogenome sequences that showed more than 82% similarity (based on Blastn) with newly sequenced mitogenome of the Eastern spinebill were included to construct genome level phylogenetic tree. Nucleotide sequences of partial cytochrome B gene were selected from the family *Meliphagidaethe* and used to align with MAFTT L-INS-I algorithm implemented in Geneious (version 7.388) (Katoh and Standley 2013). Phylogenetic analyses were performed under the GTR substitution model with 1000 bootstrap support in Geneious (version 10.2.2) and CLC Genomics Workbench (version 9.5.4). The calculations of sequence similarity percentages were performed using tools available in Geneious (version 10.2.2).

## 3. Results

### 3.1. Eastern spinebill mitogenome structure

The overall genome architecture of the mitogenome sequence of Eastern spinebill was mostly conserved in comparison to other mitogenome sequences under the family *Meliphagidaethe*.

It had a circular genome of 16,614 bp, containing 13 methionine-initiated protein-coding genes (PCGs), two rRNA genes, 22 tRNA genes, and a control region (D-loop). The percentage of A, T, C, and G were 29.2%, 23.9%, 31.9%, and 15.1%, respectively. The AT and GC content of this complete mitogenome was 53.0% and 47.0%, respectively. The proportion of coding sequences with a total length of 11,405 bp (68.65%), which encodes more than 3801 amino acids, and all protein-coding genes started with Met. The lengths of 12S and 16S ribosomal RNA were 982 bp and 1605 bp, respectively.

### 3.2. Evolutionary relationship of mitogenome of the Eastern spinebill to other passerine species

**Figure 1.**
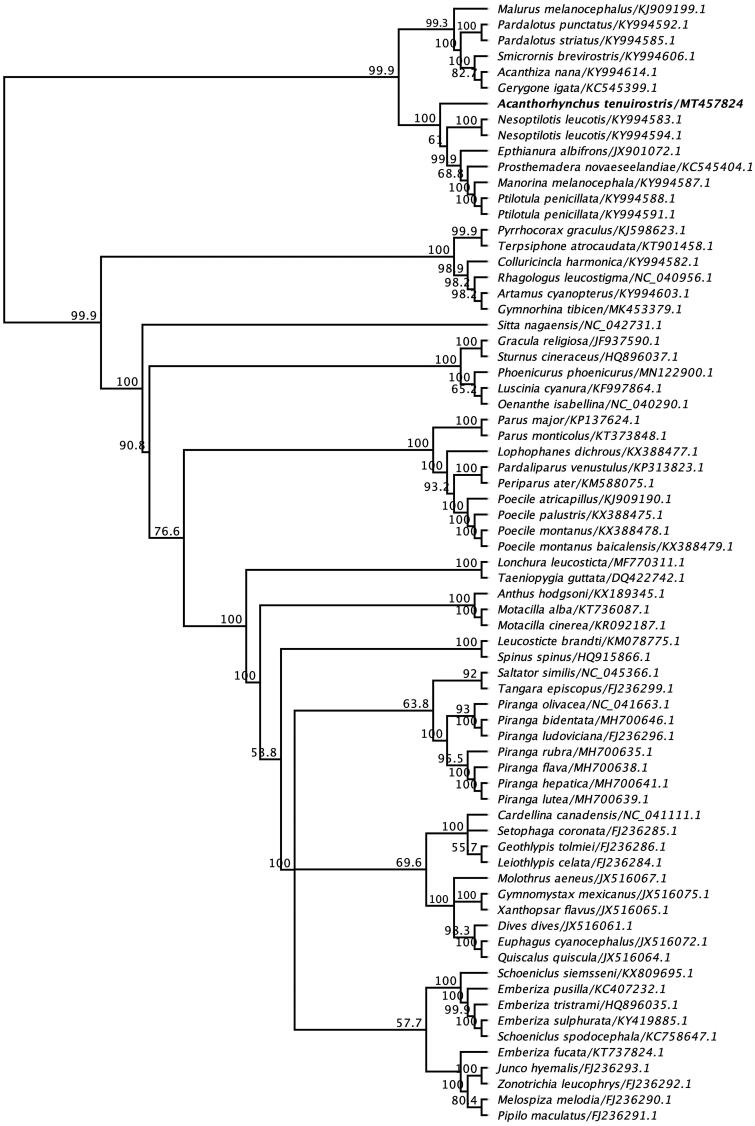
Maximum-likelihood phylogenetic tree to infer host-phylogeny relationship using complete mitochondrial genome sequenced from *A. tenuirostris* along with other selected species under the order of *Passeriformes*. The new complete mitogenome of *A. tenuirostris* was highlighted by bold font.

To understand the evolutionary relationship of the newly sequenced mitogenome of the Eastern spinebill to other passerine species, phylogenetic analysis was conducted with the inclusion of other selected complete mitogenome sequences under the order Passeriformes. In the resulting maximum-likelihood (ML) tree, mitogenome of the Eastern spinebill was placed in a well-separated monophyletic clade between other five representative species of the order *Passeriformes* (bootstrap support 100%) (Figure 1). Based on this analysis the Eastern spinebill is the sister taxon of white-eared honeyeater (*Nesoptilotis leucotis*) (GenBank accession numbers KY994583.1 and KY994594.1) found in Australia. In agreement with phylogenetic relationships, the mitogenome of the Eastern spinebill sequence demonstrated much higher sequence identities with mitogenome sequences of white-eared honeyeater (*Nesoptilotis leucotis)* and white-plumed honeyeater (*Ptilotula penicillate)* (84.9% and 85.5%, respectively).

**Figure 2.**
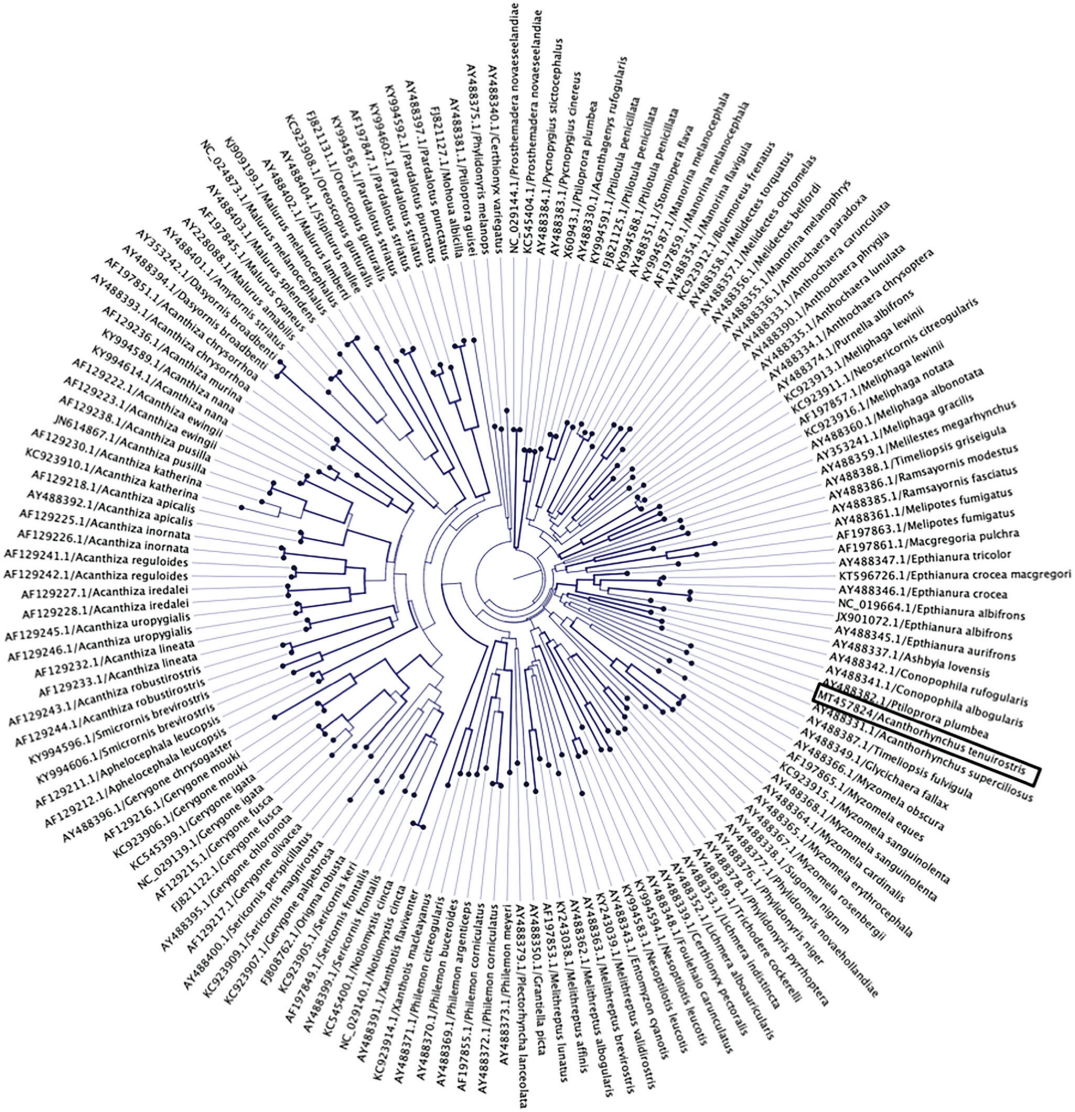
Maximum-likelihood phylogenetic tree to infer host-phylogeny relationship using partial cytochrome B gene sequenced from *A. tenuirostris* along with other selected species under the family of *Meliphagidaethe*. The *A. tenuirostris* species was highlighted by black box. The tree is displayed as circular phylogram and the labels are aligned. Bootstraps support higher 80% are highlighted by line width.

By also building phylogenetic trees using selected partial nucleotide sequences of the cytochrome B gene (Figure 2) from *Meliphagidae* family, we observed that the Eastern spinebill was placed in a sister clade with olive straightbill (*Timeliopsis fulvigula*) (GenBank accession no. AY488387) and western spinebill (*Acanthorhynchus superciliosus*) (GenBank accession no. AY488331), which is consistent with the previous studies that examined the molecular phylogenetic relatiohship among Australian passerines birds species using partial mitochondrial and nuclear genome sequences (Driskell and Christidis 2004; Gardner et al. 2010; Andersen et al. 2014).

## 4. Discussion

This paper describes the characterization and phylogenetic relationship of a novel mitochondrial genome sequenced from an Eastern spinebill. Phylogenetic analysis using the mitogenome of the Eastern spinebill positioned it among the other five representative species of the order *Passeriformes*, which has shown strong bootstrap support (100%) (Figure 1), but the Eastern spinebill remains sister to the representative clade. As shown in Figure 1, it is evident that the mitogenome of *A. tenuirostris* is most closely related to *Nesoptilotis leucotis* (84.9%), inferring that these species likely share the common evolutionary history. A well-supported phylogenetic tree was also constructed using partial nucleotide sequences of cytochrome B gene, and they showed that the Eastern spinebill was more closely related with other two species, olive straightbill and western spinebill within the family *Meliphagidae* and was placed in the same subclade (Figure 2), which is consistent with the previous findings (Driskell and Christidis 2004; Gardner et al. 2010; Andersen et al. 2014).

The spinebills, *Acanthorhynchus*, which are perhaps the most obviously specialized nectarivores in the family *Meliphagidae* and inhabit dry heathland and woodlands (Driskell and Christidis 2004), have no complete mitochondrial genome from their close relatives. Therefore, the well-defined phylogenetic relationship of the species is equivocal. Our findings demonstrated that *A. tenuirostris* species appear to be sister to remaining Meliphagidae species such as white-eared honeyeater (*Nesoptilotis leucotis*), white-fronted chat (*Epthianura albifrons*), tui (*Prosthemadera novaeseelandiae*), noisy miner (*Manorina melanocephala*) and white-plumed honeyeater (*Ptilotula penicillate)* (Figure 1). There were many key nodes resolved in the recent phylogenetic trees using unconserved elements of about 30% of the honeyeater species (Andersen et al. 2019); however, ample work remains to be done before achieving a solid phylogenetic framework of the honeyeaters. Particularly, it will be important to make availability of the genome-level data sets for the comparative studies of the morphological, behavioral and biogeographical diversification of the honeyeaters.

## Data Availability

Raw sequencing datasets that support the findings of this study are accessible through Mendeley Data repository via the following Link (http://dx.doi.org/10.17632/57zhhhs7xz.1) (Sarker 2021).

## References

[CIT0001] Andersen MJ, McCullough JM, Friedman NR, Peterson AT, Moyle RG, Joseph L, Nyári ÁS. 2019. Ultraconserved elements resolve genus-level relationships in a major Australasian bird radiation (Aves: Meliphagidae). Emu - Austral Ornithol. 119(3):218–232.

[CIT0002] Andersen MJ, Naikatini A, Moyle RG. 2014. A molecular phylogeny of Pacific honeyeaters (Aves: Meliphagidae) reveals extensive paraphyly and an isolated Polynesian radiation. Mol Phylogenet Evol. 71:308–315. 24315868 10.1016/j.ympev.2013.11.014

[CIT0003] Bankevich A, Nurk S, Antipov D, Gurevich AA, Dvorkin M, Kulikov AS, Lesin VM, Nikolenko SI, Pham S, Prjibelski AD, et al. 2012. SPAdes: a new genome assembly algorithm and its applications to single-cell sequencing. J Comput Biol. 19(5):455–477. 22506599 10.1089/cmb.2012.0021 PMC3342519

[CIT0004] Driskell AC, Christidis L. 2004. Phylogeny and evolution of the Australo-Papuan honeyeaters (Passeriformes, Meliphagidae). Mol Phylogenet Evol. 31(3):943–960. 15120392 10.1016/j.ympev.2003.10.017

[CIT0005] Gardner JL, Trueman JW, Ebert D, Joseph L, Magrath RD. 2010. Phylogeny and evolution of the Meliphagoidea, the largest radiation of Australasian songbirds. Mol Phylogenet Evol. 55(3):1087–1102. 20152917 10.1016/j.ympev.2010.02.005

[CIT0006] Katoh K, Standley DM. 2013. MAFFT multiple sequence alignment software version 7: improvements in performance and usability. Mol Biol Evol. 30(4):772–780. 23329690 10.1093/molbev/mst010 PMC3603318

[CIT0007] Keast A. 1985. An introductory ecological biogeography of the Australo-Pacific Meliphagidae. N Z J Zool. 12(4):605–622.

[CIT0008] Lea AH, Gray JT. 1936. The food of Australian birds: an analysis of the stomach contents. Emu. 35(3):251–280.

[CIT0009] Sarker S. 2021. Metagenomic Dataset of an Eastern spinebill (*Acanthorhynchus tenuirostris*) (Mendeley).

[CIT0010] Sarker S, Batinovic S, Talukder S, Das S, Park F, Petrovski S, Forwood JK, Helbig KJ, Raidal SR. 2020. Molecular characterisation of a novel pathogenic avipoxvirus from the Australian magpie (*Gymnorhina tibicen*). Virology. 540:1–16. 31726310 10.1016/j.virol.2019.11.005

[CIT0011] Sarker S, Das S, Lavers JL, Hutton I, Helbig K, Imbery J, Upton C, Raidal SR. 2017. Genomic characterization of two novel pathogenic avipoxviruses isolated from pacific shearwaters (*Ardenna* spp.). BMC Genomics. 18(1):298. 28407753 10.1186/s12864-017-3680-z PMC5390406

[CIT0012] Sarker S, Isberg SR, Milic NL, Lock P, Helbig KJ. 2018. Molecular characterization of the first saltwater crocodilepox virus genome sequences from the world's largest living member of the Crocodylia. Sci Rep. 8(1):5623. 29618766 10.1038/s41598-018-23955-6 PMC5884845

[CIT0013] Sarker S, Roberts HK, Tidd N, Ault S, Ladmore G, Peters A, Forwood JK, Helbig K, Raidal SR. 2017. Molecular and microscopic characterization of a novel *Eastern grey kangaroopox virus* genome directly from a clinical sample. Sci Rep. 7(1):16472. 29184134 10.1038/s41598-017-16775-7 PMC5705601

[CIT0014] Sarker S, Sutherland M, Talukder S, Das S, Forwood JK, Helbig K, Raidal SR. 2019a. The first complete mitogenome of Indian ringneck (*Psittacula krameri*) demonstrates close phylogenetic relationship with Eclectus parrot. Mitochondrial DNA B. 4(2):3579–3581. 10.1080/23802359.2019.1676676 PMC7707189 33366094

[CIT0015] Sarker S, Talukder S, Sutherland M, Forwood JK, Helbig K, Raidal SR. 2019b. Characterization of the first mitochondrial genome of a little Corella (Cacatua sanguinea) and its phylogenetic implications. Mitochondrial DNA B. 4(2):3792–3794. 10.1080/23802359.2019.1682481 PMC7707406 33366194

